# Burden of invasive pneumococcal disease in children in Casablanca, Morocco four years after the introduction of pneumococcal vaccination

**DOI:** 10.11604/pamj.2022.41.2.29449

**Published:** 2022-01-03

**Authors:** Amar Chikhaoui, Néhémie Nzoyikorera, Idrissa Diawara, Zineb Jouhadi, Khalid Zerouali

**Affiliations:** 1 Faculty of Medicine and Pharmacy of Casablanca, Hassan II University of Casablanca, Casablanca, Morocco,; 2 Bacteriology, Virology and Hospital Hygiene Laboratory, Ibn Rochd University Hospital, Casablanca, Morocco,; 3 Faculty of Sciences and Health Techniques, Mohammed VI University of Health Sciences (UM6SS), Casablanca, Morocco,; 4 Infectious Diseases Department, Children's Hospital, Ibn Rochd University Hospital, Casablanca, Morocco

**Keywords:** *Streptococcus pneumonia*, invasive pneumococcal disease, pneumococcal conjugate vaccine, children

## Abstract

Streptococcus pneumonia is a common bacterium that can cause several types of infections, including invasive infections especially in children aged <5 years. The aim of this work is to report the different aspects of invasive pneumococcal disease (IPD) in a pediatric hospital in Casablanca, Morocco 4 years after the implementation of pneumococcal vaccination. We conducted a descriptive, retrospective study over a 4-year period from January 2015 to December 2018 in A. Harouchi Pediatric Hospital in Casablanca. This study included hospitalized children aged 0 to 14 years´ old who had an IPD. The data was collected using a data collection sheet from archived patient records and computerized laboratory database; organization of data was done using Microsoft Excel 2016 and analysis was done using SPSS-20. A total of 68 patients were included in this series over the 4-year period. Meningitis was the most common IPD (54.41%) followed by bacteremia (19.17%) and then pneumonia (16.17%). Of the 35 serogrouped strains, 7 were included in the pneumococcal conjugate vaccine (PCV) 10 (20%), 6 were PCV13-nonPCV10 serotypes (17.14%) and 6 were non-vaccine serotypes (17.14%). Among the strains tested for their antibiotic resistance profile, 32.70% were resistant to penicillin, tetracycline (29.78%), erythromycin (20.75%) and cotrimoxazole (17.31%). One strain was intermediate to ceftriaxone. The evolution was unfavorable for 18 patients (26.47%). This study reported high resistance rates to penicillin, tetracyclin and erythromycin. The mortality essentially concerned meningitis patients. Ongoing surveillance of antibiotic susceptibility and serotype distribution is needed by a national surveillance network.

## Introduction

*Streptococcus pneumonia* (*S. pneumonia*), commonly known as pneumococcus, is a common bacterium of the human nasopharynx. Pneumococcal colonization begins in the first few months of life [1]. It becomes common in the early years and decreases with age [1]. Depending on the predisposing factors of the host and the virulence factors of the germ, *S. pneumonia* can cause a wide spectrum of diseases ranging from invasive infections to less severe but more frequent illnesses [1]. The highest rate of these infections occurs in infants, children under 2 years old and the elderly [1]. Non-invasive infections can be non-bacteremic pneumonia, otitis media or sinusitis, while the most frequent invasive infections are meningitis, bacteremia and bacteremic pneumonia. Besides, pneumococcus can cause other infections but less frequently such as peritonitis, dermo-hypodermatitis and arthritis [2].

The introduction of pneumococcal vaccines has led to a worldwide reduction in the incidence of pneumococcal infections caused by vaccine serotypes and a reduction in resistant strains of pneumococcus; however, it has also led to an increase in pneumococcal infections caused by non-vaccine serotypes (serotype replacement) [3,4]. Despite an overall decline in their incidence, pneumococcal diseases remain a major public health problem worldwide, particularly in developing countries [5]. According to the World Health Organization (WHO) in 2015, of the global deaths among <5 years old children estimated at 5.83 million, 294,000 deaths were caused by pneumococcal infections [5]. Regarding morbidity, 3.7 million episodes of severe pneumococcal infections were recorded in 2015 in children under 5 years of age whereas 630,000 cases of patients with disabilities caused by pneumococcal meningitis were recorded in 2016 [4,6,7].

Out of the 145 countries that have introduced PCV, 77% use the PCV-13 and 19% use the PCV-10, as is the case of Morocco [8]. It should be noted that very few countries have “retrograded” from PCV-13 to PCV-10, these countries include Belgium, New Zealand, El Salvador, Quebec and some regions in Sweden and Italy [9]. Concerning vaccination, PCV-13 was introduced in the Moroccan national immunization program in October 2010 and replaced by the PCV-10 in July 2012 for financial reasons [3]. The actual schedule is 2+1 at 2, 4 and 12 months of age. Following the PCV introduction, a significant decrease in invasive pneumococcal disease (IPD) was observed, essentially for < 2 years old [3]. A study was done by Diawara *et al*. at the Ibn Rochd University Hospital of Casablanca comparing the incidence rate of IPD, the rate of antibiotic resistance and serotype distribution among children ≤ 5 years old before and after PCVs introduction (January 2007 to October 2010 and from January 2011 to December 2014, respectively) in Casablanca, Morocco [3].

Results showed a great decrease in incidence rate of IPD in children ≤ 2 years of age declining from 34.6 to 13.5 per 100,000 populations before and after vaccination, respectively. The incidence rate of PCV-7, PCV-10 non-PCV-7 and PCV-13 non-PCV-10 serotypes decrease significantly from 18.0 to 4.6, from 5.7 to 1.3 and from 5.7 to 0.8/100,000 population (p < 0.001) in the same age, respectively. A significant decrease of penicillin and cotrimoxazole non-susceptible strains was also observed [3]. This present work aims to report the epidemiological, clinical, bacteriological and evolutionary aspects of invasive pneumococcal infections through a study carried out at Casablanca´s A. Harouchi Pediatric Hospital during the period from 2015 to 2018. Furthermore, the objective of this work is also to evaluate the impact of the pneumococcal vaccination in children in Casablanca in the late post-PCV period.

## Methods

**Study design:** this study is a descriptive and retrospective review of all laboratory confirmed IPD cases in A. Harrouchi Pediatric Hospital from January 2015 to December 2018. Data collection was done with the help of a data collection sheet from different sources: socio-demographic, epidemiological, clinical and evolutionary information were collected from the non-computerized archives of medical records and hospitalization registers of the different pediatric departments; biological, bacteriological and serotypes data was collected from the computerized database of the microbiology laboratory; causes of death were collected from the Professional Affairs Department of the Children's Hospital after consulting the death certificates of each deceased patient.

**Study population:** this study was carried out at A. Harouchi Pediatric Hospital in Casablanca - which is part of the Ibn Rochd University Hospital in Casablanca. It´s a tertiary hospital covering the entire region of Grand Casablanca. A. Harouchi Pediatric Hospital comprises 240 beds and manages approximately 20,000 hospitalizations each year. All children aged < 15 years old diagnosed with confirmed IPD between the 1^st^ January 2015 and 31^st^ December 2018 at A. Harouchi Pediatric Hospital were retrospectively enrolled in this study.

**Isolate collection:** all duplicate samples isolated from the same site for the same patient and all respiratory samples taken secondarily to the admission diagnosis were excluded. The diagnosis of IPD was based on positive cultures and/or polymerase chain reaction (PCR) samples of *S. pneumonia* that were isolated from normally sterile body sites (cerebrospinal fluid (CSF), blood, pleural fluid, peritoneal fluid, ascites and pus sample of mastoiditis). *S. pneumonia* isolates were identified following standard procedures of bacteriology (i.e. Î±- hemolysis, optochin susceptibility and bile solubility).

**Antimicrobial testing:** antimicrobial susceptibility testing was done following the European Committee on Antimicrobial Susceptibility Testing (EUCAST) recommendations. Erythromycin, tetracyclin, chloramphenicol, trimethoprim-sulfamethoxazol (co-trimoxazol), levofloxacin, rifampicin, vancomycin were tested by disk diffusion with antibiotic disks from Oxoid (Basingstoke, United Kingdom) on Mueller Hinton Agar supplemented with 5% sheep blood (BioMerieux, Marcy l´Étoile, France). Oxacillin (1 µg) was used for screening of penicillin non-susceptible *S. pneumonia* (PNSP). A minimal inhibitory concentration (MIC) for penicillin and ceftriaxon was determined on 5% sheep blood Mueller Hinton agar with E-tests from Oxoid (Oxoid, Basingstoke, UK). The breakpoints used for interpretation were those recommended by the EUCAST depending on the year in which the samples were taken.

**Serogrouping/serotyping of the pneumococcal isolates:** serogrouping was done by the checkerboard method with Pneumotest-latex (Statens Serum Institute Antisera, Copenhague, Denmark) and serotyping was performed by Quellung capsule swelling using Statens Serum Institute Antisera (Copenhague, Denmark). Serogrouping / serotyping was done by multiplex PCR described previously by CDC (Centers for Disease Control and Prevention) [10].

**Data analysis:** study data was entered on Microsoft Excel 2016 from the hard copies of the study collection sheets. Discrepancies were resolved by re-verification of the hospitalization registers and archived medical records. For analysis of serotype distribution, serotypes were categorized as PCV7 types (4, 6B, 9V, 14, 18C, 19F, and 23F); PCV10 types (PCV7+ 1, 5, and 7F); PCV13 (PCV 10 + 3,6A, and 19A); and non-vaccine serotypes. The epidemiological, clinical and laboratory data were analyzed using SPSS-20. Variables were expressed as numbers and/or percentages.

**Ethical considerations:** consent was obtained from the hospital heads of clinical services to publish the data and patient anonymity was maintained.

## Results

**Figure 1:**
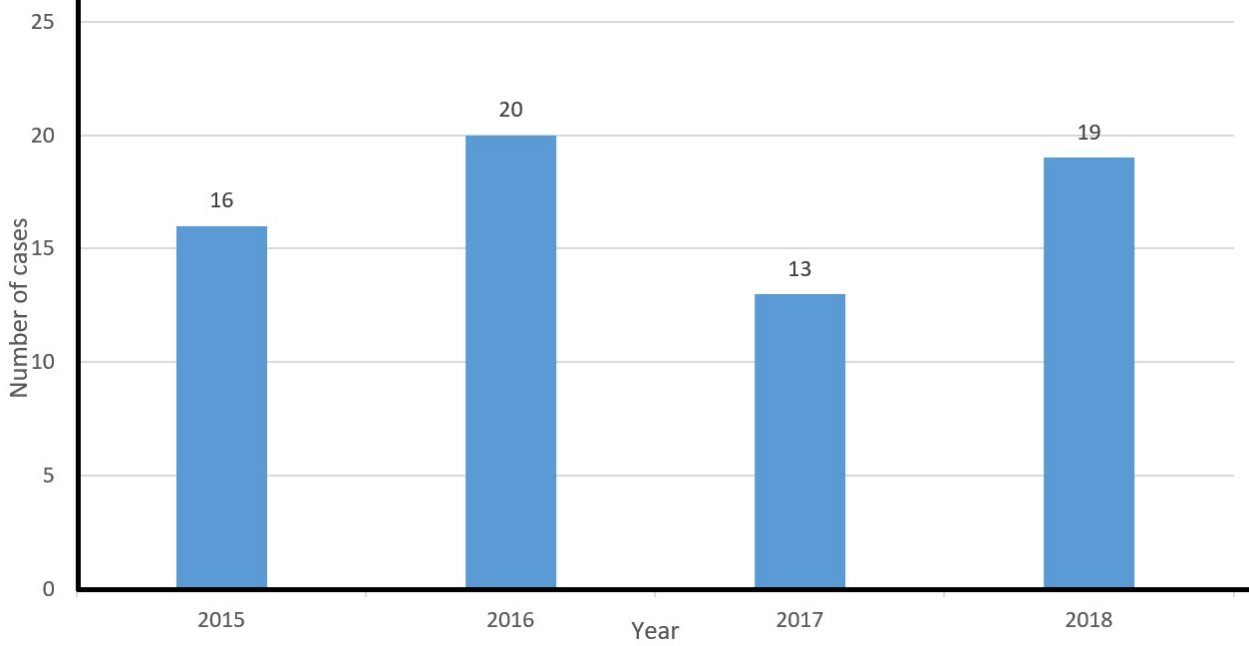
yearly distribution of pediatric IPD cases

**Socio-demographic data:** taking inclusion and exclusion criteria into account, 68 patients with IPD were identified. It is to be noted that 1 patient had 2 IPDs in 2015 and 2017 but was counted as 1 in the total number of cases. Main socio-demographic findings are described in Table 1. The patients´ mean age was 14 months and 37 patients (54.41%) were less than 2 years old; of them, 84% were less than 1-year-old, representing 45% of the total number of patients. Thirty nine patients (57.35%) were male with a M/F sex-ratio of 1.34. Only 1 patient was a neonate. A seasonal trend was observed, with 70.58% of cases diagnosed during autumn and winter. A peak of cases was observed in 2016 (Figure 1). Socio-economic status was low for 67.64% (n=46) patients.

**Table 1:** epidemiological and clinical characteristics of pediatric IPD patients

Characteristics	Number of patients (n)	Percentage (%)
**Age groups**		
0 - 2 years	37	54.41
2 - 5 years	12	17.65
5 - 14 years	16	23.53
Unknown	3	4.41
**Sex**		
Female	29	42.65
Male	39	57.35
**Seasonal distribution**		
Autumn	26	38.23
Winter	22	32.35
Spring	14	20.60
Summer	6	8.82
**Socio-economic status**		
Low	46	67.65
Middle	20	29.41
Unknown	2	2.94
**Vaccination**		
Complete according to age	27	39.71
Non-eligible (<2 months of age)	8	11.76
Born before PCV introduction	7	10.30
Mixed vaccination (PCV-10 and PCV-13)	2	2.94
Absent vaccination	1	1.47
Unknown	23	33.82
**Concomitant illnesses**		
Nephrotic syndrome	2	2.94
Lupus	1	1.47
Pulmonary tuberculosis	1	1.47
Hemorrhagic stroke	1	1.47
Pancytopenia	1	1.47
B-cell acute lymphoblastic leukemia (relapse)	1	1.47
Hematemesis (esophageal varices)	1	1.47
Hemorrhagic syndrome	1	1.47

**Medical history of the patients:** in our series, 51.47% (n=35) patients had 1 or more siblings while 23.52% (n=16) had none. The use of antibiotics prior to hospitalization was noted in 16.17% (n=11) of the patients. Regarding vaccination, 29 (42.64%) patients were fully vaccinated according to their age, 2 (2.94%) had a mixed vaccination: 2 doses of PCV-13 and 1 dose of PCV-10. More details are given in Table 1.

**Clinical presentations:** nine (13.23%) patients had concomitant illnesses, of which 2 had a nephrotic syndrome. All concomitant illnesses are described in Table 1. Meningitis was observed in 37 (54.41%) cases, bacteremia in 13 (19.17%) cases, pneumonia in 11 (16.17%) cases and peritonitis in 5 (7.35%) cases. It should be noted that 1 case of multi-bacterial appendicitis (*S. pneumoniae, E. coli* and beta-hemolytic group F *Streptococcus*) was observed and also 1 case of acute otitis media complicated by mastoiditis.

**Bacteriological data:** a total of 83 *S. pneumonia* strains was isolated from 68 patients, including 40 isolates from blood, 34 from CSF, 5 from ascites, 2 from pleural fluid and 1 each for peritoneal fluid and ear pus sample. Fifteen patients (22.05%) had repeated isolates from different types of specimens (13 repeated isolates from blood and CSF and 2 repeated isolates from blood and ascites).

**Antimicrobial profile:** antimicrobial testing was performed on 55 (80.88%) strains. Fifty two strains were tested for penicillin-susceptibility: 17 (32.69%) were resistant. Of the 53 tested strains, 20.75 % (n=11) were resistant to erythromycin. Concerning tetracyclin, 29.78% (n=14) of the 47 tested strains were resistant. Furthermore, 52 strains were tested for co-trimoxazol susceptibility: 17.31% (n=9) were resistant. All antimicrobial testing findings are described in Table 2.

**Table 2:** antimicrobial testing of strains of pediatric IPD cases

Antibiotic	Tested strains	Susceptible	Intermediate susceptibility	Resistant
Penicillin	52	35 (67.30%)	-	17 (32.70%)
Ceftriaxon	25	24 (96%)	1 (4%)	-
Erythromycin	53	41 (77.36%)	1 (1.89%)	11 (20.75%)
Levofloxacin	46	45 (97.83%)	-	1 (2.17%)
Tetracyclin	47	30 (63.82%)	3 (6.40%)	14 (29.78%)
Vancomycin	41	41 (100%)	-	-
Co-trimoxazol	52	42 (80.77%)	1 (1.92%)	9 (17.31%)
Chloramphenicol	44	42 (95.45%)	-	2 (4.55%)
Rifampicin	17	17 (100%)	-	-

**Serotype distribution:** serotyping was done on 35 (51.47%) strains (Table 3). The most common serogroups/serotypes were 1, 19A and 6. Of these serotypes, 7 (20%) are included in the PCV-10 while 6 (17.14%) were PCV-13-non PCV-10 serotypes (3, 6A and 19 A), which makes 13 (37.14%) the number of serotypes included in the PCV-13. However, 6 (17.14%) were categorized non-vaccine type strains.

**Table 3:** isolated pneumococcal serotypes from pediatric IPD cases

Serogroup	Serotype	Number of isolates	
1	-	4	4
3	-	2	2
4	-	1	1
5	-	1	1
6	Not serotyped	2	3
	D	1	
9	V/A	1	2
	N/L	1	
11	-	1	1
14	-	1	1
15	Not serotyped	1	2
	B/C	1	
17	F	1	1
18	-	2	2
19	A	4	4
dNon-typable	-	11	11
Total	-	35	35

**Outcome of the patients:** complications were observed in 34 (50%) patients (Table 4), the most common was status epilepticus for 10 patients. Seven patients had more than 1 complication. Eighteen deaths were recorded (case fatality rate of 26.47%): 10 of these patients were diagnosed with meningitis. The M/F sex-ratio of the deceased patients was 1. Of these patients, 15 (83.33%) were aged < 5 years old while 11 (61.11%) patients were < 2 years old.

**Table 4:** complications observed in pediatric IPD cases

Complication	Number of patients (n)	Percentage (%)
Status epilepticus	10	14.70
Septicemia	8	11.76
Cerebral empyema	6	8.82
Pleurisy	5	7.35
Hydrocephalus	4	5.88
Cerebral ischemia	3	4.41
Consciousness disorders	3	4.41
Purpura fulminans	2	2.94

## Discussion

More than half (54.41%) the patients were aged < 2 years old and 45% were aged < 1 year old. These findings are consistent with other studies: children <2 years old represented 49%, 80% and 55% in the series done in China, Algeria and Tunisia, respectively [11-13]. In our series, 1 neonatal case was reported. Neonatal IPD is characterized by its rarity and high mortality rate, as reported in a South African study published in 2019 which reported 4.5% neonatal cases out of 6583 IPD cases in children < 2 years old; the mortality rate was 31% [14]. Most patients (54.41%) were diagnosed with meningitis, which is similar to what was reported in the European Center for Disease Prevention and Control (ECDC) report for children < 1 year old and 4-15 years old [15]. However, in this same report, children aged 1-4 years old were diagnosed mostly with septicemia and bacteraemic pneumonia. Levy *et al*. and Al-Jardani *et al*. also reported pneumonia as the most frequent diagnosis (31% and 52.3%, respectively) [16,17]. The explanation for this difference could be the fact that, in Morocco, not all patients diagnosed with pneumonia get blood cultures. Furthermore, a lot of pneumonia cases are treated on an outpatient basis.

It should be noted that 1 appendicitis case was reported in our study. Diagnosis of pneumococcal appendicitis is very rare (0.3%) and it occurs mainly in the presence of predisposing conditions such as HIV, splenectomy and hemophilia. As is the only case in our series, appendicitis in children is typically poly microbial [18]. Relating to antimicrobial resistance, 32.70% of tested strains were resistant to penicillin in our series. This is slightly more than what was reported by Diawara *et al*. in 2015 in Casablanca (21.9% for < 2 years old and 30.8% for 2 - 5 years old) [3]. The same observation was made for co-trimoxazol and chloramphenicol.

Iroh Tam *et al*. reported 22% of strains resistant to penicillin in a meta-analysis concerning 21 African countries [19]. It should be noted that resistance rates to penicillin varied greatly between regions in this study (<50% penicillin susceptibility in Northern and Southern Africa, 95.5% in Western and Central Africa). Besides, the ECDC reported a 3% resistance rate to penicillin [15], which is significantly lower than in our study; a 2% resistance rate to cephalosporins was also reported, which is consistent with findings from Iroh Tam *et al*. [19]. It is worth mentioning that no resistance to cephalosporins was found in our series, which is the molecule used in most cases of IPD.

In our series, 35 strains were serogrouped/serotyped. Thirteen (37.14%) serotypes were included in the PCV-13 of which 6 were PCV-13-non PCV-10 serotypes. Non-vaccine serotypes represented 17% (6/35). Unfortunately, the low number of serotyped strains does not allow us to make any valid comparisons or conclusions. However, it is important to note that the 4 isolated strains of serotype 19A had high levels of resistance: 2/4 were resistant to penicillin, 2/4 to tetracycline and 3/4 to co-trimoxazol. Further studies are needed to clarify if replacing PCV-10 with PCV-13 would have a significant impact on cases caused by PCV-13-non PCV-10 serotypes and on antimicrobial resistance.

The overall mortality rate was 26.47% (18 patients), which is substantially higher than reported elsewhere: France (5%) [16], China (2.44%) [11], Brazil (10%) [20], Senegal (17.4%) [21]. The ECDC reported 3% mortality rate for pediatric IPD [15]. This high mortality rate may be due to several factors. One of these factors may be the severity of the initial presentation: 4 patients died on the day of admission and 3 others died less than 2 days after admission. Most deceased patients were diagnosed with meningitis (10/18; 55.55%). The same results were found in other series: Levy *et al*. reported that 10.5% of meningitis patients died and in the case series of Ba *et al*. 87% of deceased patients presented with meningitis [16,21].

**Limitations:** as a retrospective and single-centered study, it had limitations. Pediatric IPD cases hospitalized at regional or private hospitals were not included in this study. And even though A. Harouchi Children´s Hospital is the largest pediatric center in the region of Casablanca-Settat, the most populous region of Morocco, the number of pediatric IPD in likely underestimated and the results of this study may not be representative of regional or national epidemiology. Furthermore, the limited number of cases restricts the possibility of identifying factors associated with different aspects of IPD or to clearly measure the impact of vaccination in the late post-PCV period.

## Conclusion

*S. pneumonia* is a pathogen that still causes a great number of invasive diseases, particularly in children <2 years old, many years after the introduction of PCV. The current study has revealed that diagnoses were heterogeneous but meningitis was the most frequent. Antimicrobial resistance rates remain high for penicillin and tetracycline. The mortality essentially concerned meningitis patients. Better *S. pneumonia* serogrouping and serotyping are advocated in order to better evaluate the impact of vaccination and the evolution of the serotypes causing invasive diseases.

**Recommendations:** 1) A multicenter study including all university hospital centers would provide nationally representative figures on epidemiology, evolution of antibiotic susceptibility, and serotype frequency; 2) it would be interesting for the Moroccan Health Ministry to establish a surveillance network whose mission would be to provide biological expertise, contribute to the surveillance of all aspects of pneumococcal infections (particularly antimicrobial resistances and serotype distribution) and further evaluate the impact of PCV.

**Data availability:** the data that support the findings of this study are available from the corresponding author, upon request.

### What is known about this topic

- Invasive pneumococcal disease is an important cause of morbidity and mortality in children in developing countries, particularly in Africa;
- A significant decrease in IPD was noted in Casablanca, Morocco after the introduction of PCV.

### What this study adds

- The state of IPD in the late post PCV era in Casablanca, Morocco;
- A slight increase in antibiotic resistances between early and late post PCV periods in Casablanca, Morocco;
- This study emphasizes the importance of surveillance of antimicrobial resistance and serotype distribution on a greater scale in Morocco.

## References

[1] Richter L, Schmid D, Kanitz EE, Zwazl I, Pöllabauer E, Jasinska J (2019). Invasive pneumococcal diseases in children and adults before and after introduction of the 10-valent pneumococcal conjugate vaccine into the Austrian national immunization program. PLoS One.

[2] Cohen R, Levy C, Ouldali N, Varon E (2020). Vaccins conjugués contre le pneumocoque chez l´enfant. Journal de Pédiatrie et de Puériculture.

[3] Diawara I, Zerouali K, Katfy K, Zaki B, Belabbes H, Najib J (2015). Invasive pneumococcal disease among children younger than 5 years of age before and after introduction of pneumococcal conjugate vaccine in Casablanca, Morocco. Int J Infect Dis.

[4] Wahl B, O´Brien KL, Greenbaum A, Majumder A, Liu L, Chu Y (2018). Burden of streptococcus pneumoniae and haemophilus influenzae type b disease in children in the era of conjugate vaccines: global, regional, and national estimates for 2000-15. Lancet Glob Health.

[5] World Health Organization (2019). Weekly epidemiological record vol 8 2019.

[6] Troeger C, Forouzanfar M, Rao PC, Khalil I, Brown A, Swartz S (2017). Estimates of the global, regional, and national morbidity, mortality, and aetiologies of lower respiratory tract infections in 195 countries: a systematic analysis for the global burden of disease study 2015. Lancet Infect Dis.

[7] Zunt JR, Kassebaum NJ, Blake N, Glennie L, Wright C, Nichols E (2018). Global, regional, and national burden of meningitis, 1990-2016: a systematic analysis for the global burden of disease study 2016. Lancet Neurol.

[8] International Vaccine Access Center (IVAC) Johns Hopkins Bloomberg School of Public Health (2018). VIEW-hub report: global vaccine introduction and implementation.

[9] Desmet S, Verhaegen J, Van Ranst M, Peetermans W, Lagrou K (2018). Switch in a childhood pneumococcal vaccination programme from PCV13 to PCV10: a defendable approach. Lancet Infect Dis.

[10] Centers for Disease Control and Prevention (CDC) (2019). Streptococcus lab resources and protocols.

[11] Cai K, Wang Y, Guo Z, Xu X, Li H, Zhang Q (2018). Clinical characteristics and antimicrobial resistance of pneumococcal isolates of pediatric invasive pneumococcal disease in China. Infect Drug Resist.

[12] Ziane H, Manageiro V, Ferreira E, Moura IB, Bektache S, Tazir M (2016). Serotypes and antibiotic susceptibility of streptococcus pneumoniae isolates from invasive pneumococcal disease and asymptomatic carriage in a pre-vaccination period, in Algeria. Front Microbiol.

[13] Ajmi H (2017). Burden of pneumococcal invasive diseases in a tertiary referral center for pediatrics in Tunisia. Morressier.

[14] Moodley K, Coovadia YM, Cohen C, Meiring S, Lengana S, De Gouveia L (2019). Invasive pneumococcal disease in neonates prior to pneumococcal conjugate vaccine use in South Africa: 2003-2008. Pediatr Infect Dis J.

[15] European Centre for Disease Prevention and Control (ECDC) (2019). Invasive pneumococcal disease - annual epidemiological report for 2017.

[16] Levy C, Varon E, Ouldali N, Béchet S, Bonacorsi S, Cohen R (2020). Changes in invasive pneumococcal disease spectrum after 13-valent pneumococcal conjugate vaccine implementation. Clin Infect Dis.

[17] Al-Jardani A, Al Rashdi A, Al Jaaidi A, Al Bulushi M, Al Mahrouqi S, Al-Abri S (2019). Serotype distribution and antibiotic resistance among invasive streptococcus pneumoniae from Oman post 13-valent vaccine introduction. Int J Infect Dis.

[18] Ghadage DP, Kamble DS, Nale SS, Bhore AV (2015). Appendicitis in a child due to streptococcus pneumoniae: a rare case report. J Clin Diagn Res.

[19] Iroh Tam PY, Thielen BK, Obaro SK, Brearley AM, Kaizer AM, Chu H (2017). Childhood pneumococcal disease in Africa-a systematic review and meta-analysis of incidence, serotype distribution, and antimicrobial susceptibility. Vaccine.

[20] Berezin EN, Jarovsky D, Cardoso MRA, Mantese OC (2020). Invasive pneumococcal disease among hospitalized children in Brazil before and after the introduction of a pneumococcal conjugate vaccine. Vaccine.

[21] Ba ID, Ba A, Faye PM, Thiongane A, Attiyé Kane M, Sonko A (2015). Pediatric invasive pneumococcal disease in Senegal. Med Mal Infect.

